# Virulence factors and clonal diversity of *Staphylococcus aureus* in colonization and wound infection with emphasis on diabetic foot infection

**DOI:** 10.1007/s10096-020-03984-8

**Published:** 2020-07-18

**Authors:** Kavitha Shettigar, Thokur Sreepathy Murali

**Affiliations:** 1 Department of Biotechnology, Manipal School of Life Sciences, Manipal Academy of Higher Education, Manipal, Karnataka 576104 India; 2 Present Address: Department of Medical Laboratory Technology, Manipal College of Health Professions, Manipal Academy of Higher Education, Manipal, Karnataka 576104 India

**Keywords:** Clonal diversity, Diabetic foot, Infection, *Staphylococcus aureus*, Osteomyelitis, Toxins, Virulence

## Abstract

Foot ulcer is a common complication in diabetic subjects and infection of these wounds contributes to increased rates of morbidity and mortality. Diabetic foot infections are caused by a multitude of microbes and *Staphylococcus aureus*, a major nosocomial and community-associated pathogen, significantly contributes to wound infections as well. *Staphylococcus aureus* is also the primary pathogen commonly associated with diabetic foot osteomyelitis and can cause chronic and recurrent bone infections. The virulence capability of the pathogen and host immune factors can determine the occurrence and progression of *S*. *aureus* infection. Pathogen-related factors include complexity of bacterial structure and functional characteristics that provide metabolic and adhesive properties to overcome host immune response. Even though, virulence markers and toxins of *S*. *aureus* are broadly similar in different wound models, certain distinguishing features can be observed in diabetic foot infection. Specific clonal lineages and virulence factors such as TSST-1, leukocidins, enterotoxins, and exfoliatins play a significant role in determining wound outcomes. In this review, we describe the role of specific virulence determinants and clonal lineages of *S*. *aureus* that influence wound colonization and infection with special reference to diabetic foot infections.

## Introduction

Foot ulcer is a common complication in diabetic subjects caused due to multitude of underlying risk factors including neuropathy and vascular insufficiency [1]. These open wounds favor colonization by microbes which proliferate in the wound and cause severe infection that can spread to deeper tissues thereby substantially increasing the risk of hospitalization and lower limb amputations [2]. Pathophysiology of diabetic foot infection (DFI) is complex and the wound outcome is determinant on both host factors and microbial factors including virulence [3, 4]. Diabetic foot ulcers (DFU) are quite often colonized by aerobes, anaerobes, and fungi either individually or more often as a polymicrobial community. *Staphylococcus aureus*, a major colonizer of DFU [5–7], produces abundant biofilm and thereby inhibits wound healing and exacerbates wound infection [8, 9].

*S*. *aureus* with its emerging new clones causes severe wound infection, skin and soft tissue infections (SSTI), osteomyelitis, and other unusual infections globally. Most often, *S*. *aureus* colonizes on skin or mucosal surfaces of children and HIV or diabetic patients who are more prone to *S*. *aureus* colonization [10–13]. Hospital-acquired methicillin-resistant *S*. *aureus* (MRSA) strains are largely disseminated in clinical settings and infect immunosuppressed hosts while community-associated MRSA strains can cause infections in healthy children and adults [13, 14]. Infection of mucosal surface or skin is a consequence of initial exposure eventually triggering upregulation of virulence genes [15]. *S*. *aureus* can also cause recurrent infections throughout life.

*S*. *aureus* is the predominant bacterial isolate reported from occidental countries in DFI leading to delayed wound healing. Wound adherence, persistence, and infection is enhanced by virulence factors including wide variety of enzymes and toxins elicited by *S*. *aureus* such as protease, lipases, nucleases, hyaluronidases, haemolysins (alpha, beta, gamma, and delta), and collagenase which make host tissues favorable for bacterial growth and tissue invasion. Early diagnosis and proper wound management are critical since spread of *S*. *aureus* to soft tissue and bone can significantly contribute to amputation of lower extremities [16].

Since DFU is polymicrobial nature, it is essential to consider both the microbiological and clinical features to understand microbial virulence potential of diverse microbes that cause infection and level of host susceptibility to the microbes [17, 18]. Each bacterial species differs in its virulence potential in wound environment, and it is important to evaluate the intrinsic virulence factors of isolated species to characterize and distinguish between pathogens that cause infection and colonizers [19]. In addition, it also helps to avoid misuse of antibiotics since inappropriate antibiotic usage leads to emergence of multidrug-resistant pathogens, notably MRSA. Quite often, differentiation of true infectious pathogens from colonizers is difficult especially in DFU due to the underlying risk factors of neuropathy and ischemia. In this regard, studies have been performed focusing on virulence markers and their association in wound adherence and colonization. In this review, we have focused on *S*. *aureus* virulence factors and clonal complexes commonly associated with skin and wound pathogenesis and their role in differentiating colonizing and infecting *S*. *aureus* strains in DFI.

## Search strategy and selection criteria

The relevant reference articles were identified through literature search in PubMed and Web of Science databases and were restricted to those research articles published between January 1980 and March 2020. The following descriptors were used to obtain relevant references: “*Staphylococcus aureus*,” “ulcer,” “osteomyelitis,” “infection,” “chronic wound,” “microbiota,” “virulence,” “toxins,” “molecular methods,” “clonal complexes,” “bacterial colonization,” “antimicrobial resistance,” “adherence,” “colonization,” “genetic diversity,” “gene expression,” “host factors,” “pathogenesis,” and “biofilm” in combination with the term “*Staphylococcus aureus*” and “diabetic foot” or “diabetic foot osteomyelitis” and the Boolean operators AND, OR, and NOT, in addition to truncations. We have included cohort studies, cross-sectional studies, narrative reviews, and case-control studies. Only full-text articles published in English language were included. The first screening included a review of the titles of the studies. The second screening was based on the abstracts and duplicates and articles which did not meet the eligibility criteria were excluded. The final dataset included 140 full-text articles, meeting the inclusion criteria. The identified articles were reviewed and then classified based on the study objective and were then collated to understand the role of various *S*. *aureus* virulence markers and clonal complexes in wound infections.

## DFU microbiome and altered physiopathology

Studies have reported the polymicrobial nature of DFU and the presence of large spectrum of microbes severely limits the use of traditional culture methods [7]. DFU is commonly colonized with aerobic Gram-positive cocci, facultative and obligate aerobic Gram-negative bacilli, obligate anaerobic bacteria [5, 20], and fungi [21]. The widespread occurrence of pathogenic and multidrug-resistant strains such as MRSA which express several virulence factors negatively influences treatment outcomes and leads to chronicity of ulcer. Screening of specific virulence genes and genotyping by multilocus sequence typing approach have shown that *S*. *aureus* isolates from monomicrobial and polymicrobial wounds differ in their clonal diversity and carriage of virulence genes [22]. Though infection in diabetic subjects by definition can include abscesses, necrotizing fasciitis, and osteomyelitis among many others, infected neuropathic diabetic foot ulcers remain the major problem [18]. Host factors such as neuropathy drastically reduce the sensory functions and pain perception causing ulceration which predisposes these wounds to severe bacterial infections [18]. Furthermore, it has been observed that early signs of infection can go undetected due to several underlying risk factors including reduced immunological functions [23], and if left untreated, the infection spreads to deeper tissues including bones. In diabetic subjects, impaired wound healing due to an increase in acute inflammatory cells, an absence of cellular growth, and decreased epidermal cell migration have been observed. In addition, the impaired host responses can shift the equilibrium from colonizers to pathogenic species leading to chronic non-healing wound ulcers.

## Diabetic foot osteomyelitis

Osteomyelitis is an inflammatory condition resulting from infection of bone and is commonly missed or underdiagnosed in patients with underlying diabetic foot ulcer complications. Reports suggest that 60% of DFUs get infected and 10–15% of the infected wounds usually develop into osteomyelitis [24]. *S*. *aureus* is the primary pathogen associated with diabetic foot osteomyelitis (DFOM) and results in substantial morbidity and mortality. Studies indicate that *S*. *aureus* can form biofilms on healthy bones and infect both osteoblasts and osteoclasts while both in vivo and in vitro studies clearly show that they can also replicate and proliferate inside osteoclasts and evade destruction by immune cells [25, 26]. Interestingly, even though antibodies for various *S*. *aureus* antigens (coagulase, lukD, lukE, fibronectin-binding protein, etc.) are produced in healthy individuals, *S*. *aureus* overcomes protective immune responses and causes recurrent infections by producing pathogenic antibodies that can drastically overcome adaptive immunity [27, 28]. However, genome sequencing of two *S*. *aureus* strains collected longitudinally from a chronic osteomyelitis patient showed *agrC* frameshift mutations over time resulting in reduced virulence and less tissue damage [29]. Mass-based proteomics approach in a murine osteomyelitis model demonstrated that mutations in exoprotein regulatory protein *saeRS* and staphylococcal accessory regulator *sarA* attenuates virulence by downregulating virulence factor production and degradation of virulence factors respectively [30]. Víquez-Molina et al. [31] compared the prevalence of virulence genes encoding for pvl, etA, etB, and tsst in *S*. *aureus* strains in SSTI and bone infection and found no significant difference in virulence gene profiles except for higher prevalence of pvl+ strains in soft tissue infections. Even though several clonal complexes are associated with DFU colonization and infection, there are limited studies on virulence genes and clonal complexes associated with DFOM. Lattar et al. [32] performed molecular fingerprinting of *S*. *aureus* strains from patients with osteomyelitis by pulsed-field gel electrophoresis and concluded that loss of capsular polysaccharide production was the major factor associated with chronic osteomyelitis. They also showed that higher proportion of *cap5 S*. *aureus* isolates were methicillin-resistant *S*. *aureus* (MRSA) and lukS-PV/lukF-PV+ compared with *cap8* isolates [32]. Senneville et al. [33] reported bone tropism of CC398 methicillin-susceptible *S*. *aureus* clone and its significance in DFOM.

## Virulence factors of *S*. *aureus*

### α-Toxin

In skin infections, α-toxin is considered a key virulence factor of *S*. *aureus*. This pore-forming toxin consisting primarily of beta sheets is secreted by most of the *S*. *aureus* strains as a water-soluble monomer targeting the red blood cells [34–36]. The gene coding for alpha toxin *hla* was present in *S*. *aureus* strains in all the grades of wounds in DFU though some difference was observed between MRSA and methicillin-susceptible *S*. *aureus* (MSSA) strains [37, 38].

### Panton-Valentine leukocidin

Panton-Valentine leukocidin (PVL) is a potent cytotoxin that consists of two chromatographically separate protein components, namely LukS-PV (slow) and LukF-PV (fast). The active toxin causes lysis of neutrophils by forming a pore on its membrane and is associated with dermonecrosis, chronic SSTI [39, 40], recurrent mucocutaneous infections [41], and necrotizing pneumonia [42]. Further, PVL*-*carrying strains can cause chronic SSTI and necrotizing pneumonia in otherwise healthy individuals (Table 1). Though PVL-encoding strains are much less prevalent in community with < 10% MSSA clinical isolates found to encode *pvl* gene, studies indicate that isolates carrying gene coding for PVL can result in wound worsening.

**Table 1:** *S*. *aureus* virulence factors involved in wound progression

Virulence factors	Function	Role in infection	References
MSCRAMMs			
Bone sialoprotein-binding protein (isoform of SdrE) (Bbp)	Adhesion to extracellular matrix, bone and joint tissue, fibrinogen	Osteomyelitis	[43, 44]
Cap5 and Cap8	Inhibits interaction between C3b, immunoglobulin and receptors; targets phagocytes; promotes virulence in *Caenorhabditis elegans*	Mastitis, cystic fibrosis, endocarditis	[45]
Collagen adhesin (Cna)	Collagen-binding adhesin mediates binding to cartilage/ collagen-rich tissue, blocks complement activation	Osteomyelitis, septic arthritis, keratitis	[46–49]
Fibronectin-binding proteins A (FnBPA) and B (FnBPB)	FnBPA binds to fibrinogen and elastin; FnBPB binds to fibronectin; adhesion to ECM	Endocarditis, implant orthopaedic infections, osteomyelits, arthritis	[46, 48, 50]
Iron-regulated surface determinant protein H (IsdH)	Haem uptake and iron acquisition into bacterial cytoplasm	SSTI	[51]
Serine–aspartate repeat-containing protein D (SdrD)	Binds desquamated epithelial cells; nasal colonization	Bone infection	[52–55]
SdrE	Binds complement factor H; evades immune response; degrades C3b	SSTI	[56]
Bone sialoprotein-binding protein (isoform of SdrE)	SD-rich fibrinogen-binding, bone sialoprotein-binding protein	Osteomyelitis, arthritis	[57]
Toxins/superantigens			
Epidermal cell differentiation inhibitor (Edin)	Inhibits actin cytoskeleton of epithelial and endothelial barrier; formation of large transcellular tunnels; targets host Rho proteins; inhibits complement-mediated phagocytosis	Bacteremia	[58–61]
LukDE	Kills leukocytes and macrophages via chemokine receptors	Dermonecrosis	[62, 63]
PVL	Targets complement receptors C5aR and C5L2, apoptosis of neutrophils, necrosis	Necrotizing pneumonia, SSTI, furunculosis	[42, 64–67]

### Enterotoxins

**Fig. 1:**
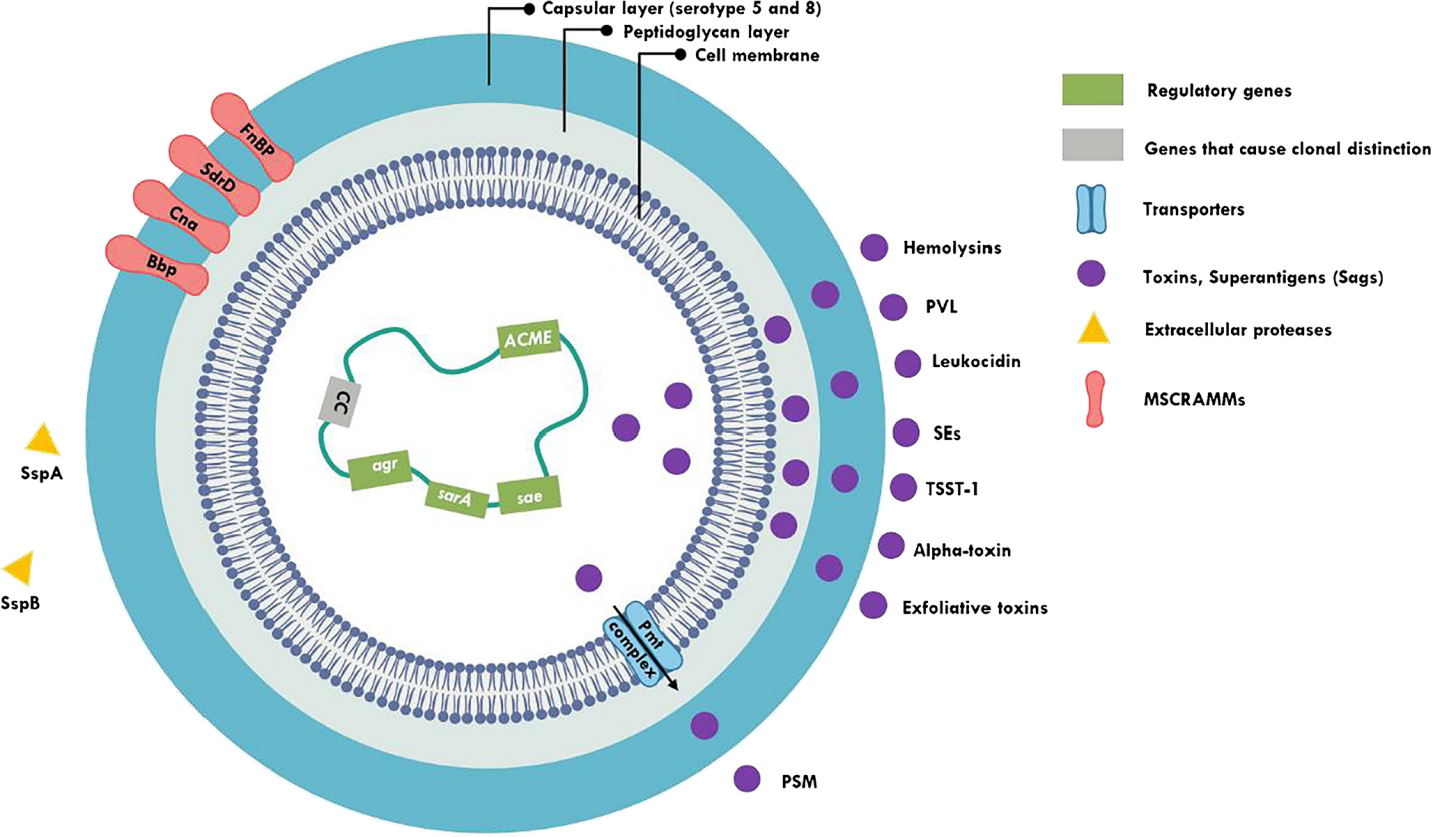
Schematic diagram illustrating major *S*. *aureus* factors associated with DFI and DFOM. (Adapted from Kong et al. [13]). ACME, arginine catabolic mobile element; agr, accessory gene regulator; Bbp, bone sialoprotein-binding protein; CC, clonal complexes; Cna, collagen adhesin; FnBP, fibronectin-binding protein; MSCRAMMs, microbial surface components recognizing adhesive matrix molecules; PMT complex, PSM transporter complex; PSM, phenol-soluble modulins; PVL, Panton-Valentine leukocidin; SAgs, super antigens; sarA, staphylococcal accessory regulator; sae, response regulator; SdrD, serine–aspartate repeat-containing protein D; SEs, staphylococcal enterotoxins; SspA, staphylococcal serine protease; SspB, cysteine protease; TSST-1, toxic shock syndrome toxin-1

*S*. *aureus* produce several exoproteins including staphylococcal enterotoxins (SEA, SEB, SECn, SED, SEE, SEG, SEH, and SEI), exfoliative toxins (ETA and ETB), and leukocidin (Fig. 1). Toxic shock syndrome toxin (TSST-1) and staphylococcal enterotoxins, collectively termed as pyrogenic toxin superantigens (PTSAgs), are known to play a significant role in proliferation of T cells irrespective of antigenic specificity. The majority of *S*. *aureus* isolates of DFU produce large number of SAgs [68], while SAg exotoxins have also been shown to contribute significantly to other major illnesses [69]. Higher number of *S*. *aureus* strains isolated from wound grades 2–4 of Wagner Classification System was shown to harbor genes encoding enterotoxins SEA and SEI than strains from grade 1 ulcer [70], making them potent markers to differentiate colonization from infection. Interestingly, *S*. *aureus* strains from DFU share more similarity with strains from atopic dermatitis and normal vaginal mucosa in their distribution and production of more types of SAgs per organism suggesting that DFU strains originated and were better adapted to skin compared with mucosae which produce fewer SAgs.

### Toxic shock syndrome toxin-1

Toxic shock syndrome toxin-1 (TSST-1), a 22-kD SAg, causes toxic shock syndrome. Another new member of the SAg family, SEI-X, is known to cause necrotizing pneumonia [58]. Both SEI-X and TSST-1 have potential role in DFU pathogenesis [68]. Even though the carriage of *tsst*-1 is low in DFU isolates, they are significantly more abundant in grade 4 ulcer than in DFOM [33].

### Epidermal cell differentiation factor

The epidermal differentiation factor (EDIN) and EDIN-like factors are a family of exotoxins that specifically inhibit host protein RhoA [59], which negatively impacts host tissue by favoring bacterial dissemination and hindering complement-mediated phagocytosis. Recent findings hypothesize the role of EDIN in disseminating between tissues by hematogenous route through intracellular tunnel formation in endothelial cells named macroapertures [60, 71]. *Edin*-positive strains were found to be more prevalent in moderate-to-severe grade DFUs than in low-grade infection. These strains were also associated with *agr*I cluster and virulence markers including genes coding for hemolysin, the *egc* cluster of enterotoxins, lukDE, intracellular adhesion proteins (*icaA*, *icaC*, and *icaD*), *cap5*, MSCRAMM (*clfA*, *clfB*, *fib*, *ebpS*, and *fnbA*), and antibiotic resistance (*tet* and *fosB*). *Edin*-positive isolates grouped to four major clonal complexes, a singleton closely associated with CC8 (*edin*-A), a singleton belonging to ST152-MSSA (*edin*-B), CC80-MRSA (*edin*-B), and mostly CC25/28 MSSA (*edin*-A). It is also reported that grade 1 ulcer infected with *edin*-positive strains led to poor wound outcome [72]. While CC25/CC28-MSSA and CC80-MRSA were significantly higher in *edin*-positive isolates, none of them grouped to colonizing strains of CC5/CC8 [72]. Association of *edin*-positive strains with other virulence markers in DFU has also been reported. Thus, EDIN coding genes can be considered potent markers to categorize *S*. *aureus* strains as colonizers or infectious as well as reliable predictors of the wound outcome.

### Accessory gene regulator

*S*. *aureus* pathogenicity is enhanced by quorum sensing (QS) mechanisms. Virulence factors essential for causing SSTI are regulated by accessory gene regulator (*agr*). Expression of several virulence determinants is known to be affected by the inhibitory activity of *agr* groups representing a form of bacterial interference [73]. A recent study reported that strains carrying *agr* were more pathogenic than those without [74].

### Arginine catabolic mobile element

Arginine catabolic mobile element (ACME), a genetic island consisting of clusters of genes, confers *S*. *aureus* the ability to colonize skin. ACME is horizontally transferred from *S*. *epidermidis*, a skin commensal [75], and encodes multiple genes, among which *arc* (arginine deiminase system) and *opp-3* (a ABC transporter) are vital in enhanced colonization. The arginine deiminase catabolizes l-arginine and by elevating skin’s pH makes it more amenable for microbial colonization [76]. Opp-3 enhances eukaryotic cell adhesion, peptide nutrient uptake, and resistance to antimicrobial peptides, thereby promoting the ability of bacteria to thrive on human skin. Thus, the acquisition of the mobile element has a potential role in disruption of skin barrier and bacterial invasion. Studies with *S*. *aureus* USA300 strain highlight the importance of ACME locus in enhancing pathogenicity and clonal dissemination. Even though, ACME has a potent role in success of USA300, its deletion has shown contradictory effect on competitive fitness in skin infection models [77, 78]. ACME *speG* and ACME *arc* genes mediate enhanced synthesis of polyamines in skin and cause clearance of *S*. *aureus* in murine skin abscess model [79]. Survival of USA300 in acidic environment is mediated by genes encoded by *arc* operon [79] and biofilm formation is enhanced by ACME *speG*-mediated polyamine tolerance [80] by upregulating genes involved in biofilm production and by increased adhesion properties, thereby favoring skin colonization, persistence, and transmission.

### Microbial surface components recognizing adhesive matrix molecules

Infection of a host commences with the pathogen binding to host surface components (fibrinogen, fibronectin, and epidermal keratinocytes). A family of staphylococcal cell wall-anchored adhesins, called MSCRAMMs (microbial surface components recognizing adhesive matrix molecules), plays a significant role in aiding attachment of *S*. *aureus* virulence proteins to bone matrix and collagen [81]. In osteoblasts, MSCRAMMs play a significant role by allowing bone invasion and formation of metabolically inactive small-colony variants, which exhibit significant phenotypic and metabolic differences from regular *S*. *aureus* isolates [82–85]. However, these *S*. *aureus* variants are relatively antibiotic resistant and hinder the treatment efficacy [86, 87]. Fibronectin-binding proteins (FnBPs) are the major staphylococcal adhesins which help in colonization of human airway epithelial cells and fibroblasts and thereby establish staphylococcal infection [88]. *S*. *aureus* FnBPs also play a critical role in orthopaedic implant-associated infections, osteomyelitis, and arthritis [82].

### Phenol-soluble modulins

Phenol-soluble modulins (PSMs) also play significant role in *S*. *aureus* skin infection [89]. PSMs are pore-forming toxins made up of a family of seven amphipathic α-helical peptides. Most of the *S*. *aureus* strains secrete PSMs [14] that provide them capacity to lyse human neutrophils, monocytes, erythrocytes, and osteoblasts [89] increasing tissue toxicity. Most pathogenic strains of staphylococci elicit different PSMβ peptides (PSMβ1 and 2), PSMα peptides (PSMα1–4), and a δ-toxin, thus contributing to staphylococcal pathogenesis and virulence [89]. PSMs themselves exhibit selective antimicrobial function and PSM-inspired peptides are reported to have considerable bactericidal activity against multidrug-resistant bacteria [90].

### Extracellular adherence protein

Extracellular adherence protein (Eap), a 45–70-kDa protein that binds to several proteins including fibronectin, is reported to be a significant marker of impaired wound healing in mouse model [91, 92]. Eap inhibits neovascularization by hindering the inflammatory cell response near the wound area. Studies indicate that Eap interferes in ICAM-1 (intercellular adhesion molecule-1)-dependent leukocyte-endothelium interactions restricting host leukocyte recruitment, thereby aiding in persistence of *S*. *aureus* in a hostile milieu in chronic wounds [93]. In contrast, Eap does not play a major role in virulence of *S*. *aureus* in skin wound infection models as well as systemic infection models, since Eap does not contribute to bacterial adherence to proteins other than ICAM-1 [93]. However, Eap does contribute to enhanced adhesion and internalization of staphylococci by keratinocytes in a FnBP-independent manner. Eap secreted by *S*. *aureus* also significantly contributes to the internalization of other pathogenic bacteria in the wound microenvironment [94].

### Biofilm factors

Biofilm production is an important strategy adopted by bacteria to colonize and infect skin tissues [95]. Though bacteria can be found in planktonic form in chronic wounds, they are most likely observed to form polymicrobial communities in biofilm matrix [96]. The presence of biofilms in non-healing wounds contributes significantly in hindering the effectiveness of antimicrobial agents and in overcoming host immunity. Bioactive compounds from biofilm communities of *S*. *aureus* and *Pseudomonas aeruginosa* have been shown to impair migration and proliferation of keratinocytes in chronic skin wounds and chronic tympanic membrane perforations [97]. In vitro studies also have shown that biofilm-conditioned media (BCM) from these two bacteria could inhibit cell proliferation while BCM derived from *S*. *aureus* was shown to reduce cell migration in keratinocytes and fibroblast cells in wound scratch assays [98]. Proteomic analysis of these media revealed several proteins linked to delayed wound healing including alpha hemolysin and epidermal cell differentiation inhibitor [97]. In other studies, loss of HEK cell viability by *S*. *aureus* BCM has been reported [98, 99]. HEKa cells treated with BCM showed upregulation of CXCL2, IL-8, DUSP1, and ATF3 genes which play a major role in inflammation and apoptosis [99].

## Clonal complexes

Staphylococci isolated from DFU have been found to be genetically diverse, resistant to many antibiotics and harbor several virulence determinants [100]. Using multilocus sequence typing, strains of *S*. *aureus* could be grouped into clonal lineages and the major clonal lineages in humans were found to belong to clonal complex (CC)1, CC5, CC8, CC9, CC12, CC15, CC22, CC30, CC45, and CC51 [101]. In DFU, CC5 methicillin-sensitive *S*. *aureus* (CC5-MSSA), CC8-MSSA, and CC15-MSSA were considered to be colonizing strains with a favorable outcome while CC45-MSSA strains were shown to cause severe infections [37, 72, 102]. In addition, CC45 and CC30 were also considered as causative clones of severe invasive infections [103, 104]. It is believed that DFU showing worsening outcome do not colonize with CC5/CC8-MSSA strains and clonality of these strains during admission and follow-up visit remain unchanged. CC25/CC28-MSSA and CC80-MRSA strains are also considered as infecting strains in DFU as these CCs were found significantly higher in *edin*-positive strains (Table 2), *edin* gene being a predictive risk marker for worsening ulcer [72]. Even though clonal lineages found associated with humans and animals generally are different, livestock-associated CC398 (LA-CC398] strain, associated with pigs, has emerged as a major human pathogen causing severe infections [129–131], ventilator-associated pneumonia [132], and wound infections [133]. CC398 is significantly associated with diabetic foot osteomyelitis (DFOM) strains and helps to differentiate DFOM from SSTI—two major complications of DFU—both of which are known to carry CC45-MSSA [33]. CC398 is distinct with the presence of hemolysins, genes that code for intracellular adhesion proteins, *cap5*, and MSCRAMM genes including *bbp*, *clfA* and *clfB* [33], *pvl* [134], and multiple classes of antimicrobial resistance genes [135] showing potent virulence in SSTI infections in humans.

**Table 2:** Clonal lineages and associated virulence markers of *S*. *aureus* in skin and wound infection

Source of sample	Major virulent factors/major findings	Prevalent genotype	Reference
SSTI	pvl	ST152, ST121, ST5, ST15, ST1, ST8, and ST88	[105]
SSTI, surgery infection, bone and joint infection, and others	CapH5, capJ5, capK5	CC5, CC8, CC97	[106]
	capH8, capI8, capJ8, and capK8	CC45	
	egc cluster	CC5, CC45	
	Absence of fnbB	ST228-I	
	Cna	ST239-III and ST45-IV	
SSTI	hla	ST239	[107]
Impetigo	eta	CC15, CC9, and ST88 (CC88)	[108]
	eta, etb	ST121	
Wound, urine, semen	egc	CC5, CC25, CC30, CC45, CC121	[109]
	etd	CC25, CC80	
	edinB	CC25, CC80, CC152	
Wounds, nares, blood, sputum, urine, and others	egc cluster	CC5, CC22, CC30, and CC228	[110]
	sed, sej, ser	CC8	
	Tst1	CC5, CC30	
Wound and respiratory samples	PVL	ST80-MRSA-IVc	[111]
Bone and joint infections	ACME	CC8-MSSA	[112]
	EtD, edinB	CC25, CC80	
	capH8, capI8, capJ8, capK8	CC7, CC12, CC15, CC30, CC45, CC59, ST80, CC88, ST96, CC101, CC121, ST239 and ST426	
	cna	CC12, CC22, CC30, CC45, CC96, CC121, ST239, and ST426	
	sasG (*S.aureus* surface protein G)	CC5, CC8, CC15, CC22, ST49, CC59, ST80, CC88, and ST96	
Invasive infections	Egc	CC5, CC25, CC30, and CC45	[113]
	Tst	CC30	
	Etd	CC25	
Invasive infections	Tst--1	CC30/CC39	[114]
SSTI, respiratory tract infections, osteomyelitis	Hla, psmα, RNAIII	ST59	[115]
	sasX (cell wall-anchored protein)	ST239-MRSA-SCCmecIII-t037	
Nasal swabs	Increased biofilm production at 0%, 0.1%, and 0.25% glucose concentrations	CC8	[116]
	Higher mortality rate; PSMα3 peptide variant with reduced immune-stimulatory and cytolytic activity	CC30	[117, 118]
	Osteo-articular infection	CC22	[119]
Community settings	agr-I	CC59	[120]
Community settings	PVL	ST1153-MSSA	[121]
Community, multiple clinical settings	pvl	ST1, ST5, ST8, ST22, ST30, ST80, ST772, ST452, ST59, ST93, CC121, and ST154	[122–125]
	pvl negative	ST239	[124]
Hospital settings	Tn6072	ST239	[126]
Hospitalized patients at risk of MRSA carriage	cna	CC1, 12, 22, 30, 45, 51, and 239	[127]
	TSST-1	CC30	
Multiple clinical samples	High level of Hla production	CC1, CC5, CC8, CC15, or CC96	[128]
	Complete absence of Hla production	CC22, CC30, CC45, CC479, CC705	

Association between presence of certain virulence genes and DFU outcome is reported in many studies. For instance, difference in the size of abscess formation in rabbit skin abscess model was attributed to different clonal lineages [136]. Different outcomes with difference in abscess diameter ranging from 5 to 7 cm (USA300, USA500, and ST80) and from 2 to 4 cm (USA400, USA1000, ST72, USA100) and almost complete absence of abscess (USA200, USA1100) were manifested by different *S*. *aureus* clonal lineages. Abscess size caused by USA300 was found to be comparable with that caused by USA100, USA200, USA400, USA1100, and ST72 strains and different from those carrying USA500, USA1000, and ST80 strains. Interestingly, though abscess formation by Panton-Valentine leukocidin (PVL)-positive USA300 and PVL-negative USA500 was comparable, the role of PVL in skin infection is thought to be limited in nature. Furthermore, neutrophil lysis activity of USA300 was shown to be significantly higher than that of other strains, and was suggested to be a major determinant of MRSA skin infection pathogenesis [136]. USA300 strains showed correlation between the expression of *psmα*, *hla*, and *agr* (with the exception of *lukS-PV*) and cause abscess, release cytokine, and lyse neutrophils lysis, whereas α-toxin and N-formylated PSMα3 peptide correlated with neutrophil lysis. Though the role of different *S*. *aureus* clonal lineages from blood stream infections is available, detailed studies on clonal types on DFI and their role in wound outcome are relatively less explored.

ST22 (CC22) is reported as a common type in DFU infections and all ST22 strains were shown to be positive for virulence factors *clfa* and *agr* I. Several less frequent clones have also been reported suggesting that diabetic patients can be an important route for dissemination of clones between hospital and community settings [100]. Sotto et al. [37] reported that foot ulcers with *S*. *aureus* strains of CC5 and CC8 showed favorable wound outcome and hypothesized that *S*. *aureus* of CC5/CC8 clones as colonizing and others as infecting clones.

## Distinguishing colonization from infection in DFU

The Infectious Diseases Society of America and International Working Group on the Diabetic Foot together have established specific clinical criteria to distinguish different grades of DFI severity [137, 138]. According to this classification, grade 1 wound is considered colonized wound while grade 2 or more is considered infection. Sotto et al. [37] screened *S*. *aureus* isolates from DFU of varying grades from 1 to 4 for various virulence genes and identified several toxins including leukocidins, enterotoxins, exfoliatins, and toxic shock syndrome toxin and reported that strains from grade 1 foot ulcer to have low prevalence of virulence genes. Further, they extended their study [70] to assess clonality and carriage of 31 highly prevalent virulence-associated genes to predict the wound outcome. Among the 31 genes screened, 10 genes (*sea*, *seb*, *sec*, *sei*, *sej*, *hlb*, *hlg*, *hlgv*, *cap5*, and *lukE*) were found to be significantly associated with strains from grade 2–4 ulcers, whereas *cap8* gene was associated with strains from grade 1 ulcers. None of the isolates from worsening wounds belonged to CC5 and CC8 indicating links between clonality and wound healing. However, no significant difference was found between infected and uninfected ulcers with regard to genes coding for PVL and exfoliatins [37]. But contrasting observation was found with reference to association of exfoliatins in different grades of DFU. Exfoliatin genes were found to be more likely in strains isolated from grade 4 ulcer compared with lower grades. In addition, their serotype distribution also varied with *eta* and *etb* being found very rarely (1.3%) or absent in most samples while *etd* (3.7%) was found in higher frequency. However, grade 1 ulcers harboring *S*. *aureus* strains carrying *etd* gene showed worsening wound outcome [72]. Post et al. [139] showed important differences in the presence of *eta* and *etb* gene in diabetic foot infection (*eta*, 13%; *etb*, 17%) and osteomyelitis (*eta*, 22% and *etb*, absent). One of the limitations found was the study was conducted solely on *S*. *aureus* isolates of monomicrobial wound type, while DFU is predominantly polymicrobial in nature.

Using a *Caenorhabditis elegans* model, Sotto et al. [70] showed that the pathogenicity of *S*. *aureus* strains in DFU grades higher than 2 were significantly more than in grade 1. Pathogenicity was assessed by the survival time of the nematode upon ingestion of *S*. *aureus* which was represented by LT_50_ and LT_100_ (time required to kill 50% and 100% of nematodes, respectively). Isolates from ulcer grades 2–4 showed LT_50_ < 2 days, whereas LT_50_ was > 3 days for isolates from grade 1 ulcer. LT_50_ of strains obtained from healing wounds was higher at the time of entry as well as follow-up while strains from non-healing ulcers had lower values. Messad et al. [140] identified genetic elements associated with prophage in *S*. *aureus* genome to promote colonization.

## Conclusion

DFUs are extremely vulnerable to bacterial infections that can result in lower limb amputations and even death. Though from a clinician’s perspective, it is important to differentiate colonization from infection, it might prove cumbersome in DFU due to the underlying effects of neuropathy and/or ischemia. The polymicrobial community in DFI further contributes to synergistic interaction between wound pathogens and induces various virulence traits and modulates host immunity and overall wound deterioration. Prompt recognition of worsening ulcers using predictive molecular markers will hence considerably help in preventing lower limb amputations. Distribution of isolates into different clonal complexes allows comparison between colonizing and infecting strains as well as determining the origin and clonality of the strains infecting wound ulcers. Detection of specific virulence encoding genes along with clonality in different grades will help us in identifying *S*. *aureus* strains that could cause severe negative wound outcome in DFI and also to avoid misuse of antibiotic therapy in uninfected wounds.

## Data Availability

Not applicable.

## References

[1] Lavery LA, Armstrong DG, Wunderlich RP, Mohler MJ, Wendel CS, Lipsky BA (2006). Risk factors for foot infections in individuals with diabetes. Diabetes Care.

[2] Richard J, Lavigne J, Sotto A (2012). Diabetes and foot infection: more than double trouble. Diabetes Metab Res Rev.

[3] Smart H, Al Ghareeb AM, Smart SA (2019). 25-Hydroxyvitamin D deficiency: impacting deep-wound infection and poor healing outcomes in patients with diabetes. Adv Skin Wound Care.

[4] Alvaro-Afonso FJ, Lazaro-Martinez JL, Papanas N (2018). To smoke or not to smoke: cigarettes have a negative effect on wound healing of diabetic foot ulcers. Int J Low Extrem Wounds.

[5] Murali TS, Kavitha S, Spoorthi J (2014). Characteristics of microbial drug resistance and its correlates in chronic diabetic foot ulcer infections. J Med Microbiol.

[6] Zubair M, Malik A, Ahmad J (2011). Clinico-microbiological study and antimicrobial drug resistance profile of diabetic foot infections in North India. Foot.

[7] Jneid J, Lavigne JP, La Scola B, Cassir N (2017). The diabetic foot microbiota: a review. Hum Microbiome J.

[8] Bowling FL, Jude EB, Boulton AJM (2009). MRSA and diabetic foot wounds: contaminating or infecting organisms?. Curr Diabetes Rep.

[9] Zhao G, Usui ML, Underwood RA (2012). Time course study of delayed wound healing in a biofilm-challenged diabetic mouse model. Wound Repair Regen.

[10] Wertheim HFL, Vos MC, Ott A (2004). Risk and outcome of nosocomial *Staphylococcus aureus* bacteraemia in nasal carriers versus non-carriers. Lancet.

[11] von Eiff C, Becker K, Machka K, Stammer H, Peters G (2001). Nasal carriage as a source of *Staphylococcus aureus* bacteremia. N Engl J Med.

[12] Lin J, Xu P, Peng Y (2017). Prevalence and characteristics of *Staphylococcus aureus* and methicillin-resistant *Staphylococcus aureus* nasal colonization among a community-based diabetes population in Foshan, China. J Diabetes Invest.

[13] Kong EF, Johnson JK, Jabra-Rizk MA (2016). Community-associated methicillin-resistant *Staphylococcus aureus*: an enemy amidst us. PLoS Pathog.

[14] Li M, Diep BA, Villaruz AE (2009). Evolution of virulence in epidemic community-associated methicillin-resistant *Staphylococcus aureus*. Proc Natl Acad Sci U S A.

[15] Novick RP (2003). Autoinduction and signal transduction in the regulation of staphylococcal virulence. Mol Microbiol.

[16] Boulton AJM, Vileikyte L, Ragnarson-Tennvall G, Apelqvist J (2005). The global burden of diabetic foot disease. Lancet.

[17] Jeffcoate WJ, Lipsky BA, Berendt AR (2008). Unresolved issues in the management of ulcers of the foot in diabetes. Diabet Med.

[18] Lipsky BA (2004). A report from the international consensus on diagnosing and treating the infected diabetic foot. Diabetes Metab Res Rev.

[19] Richard JL, Sotto A, Lavigne JP (2011). New insights in diabetic foot infection. World J Diabetes.

[20] Smith K, Collier A, Townsend EM (2016). One step closer to understanding the role of bacteria in diabetic foot ulcers: characterising the microbiome of ulcers. BMC Microbiol.

[21] Chellan G, Shivaprakash S, Ramaiyar SK (2010). Spectrum and prevalence of fungi infecting deep tissues of lower-limb wounds in patients with type 2 diabetes. J Clin Microbiol.

[22] Shettigar K, Jain S, Bhat DV (2016). Virulence determinants in clinical *Staphylococcus aureus* from monomicrobial and polymicrobial infections of diabetic foot ulcers. J Med Microbiol.

[23] Brem H, Tomic-Canic M (2007). Cellular and molecular basis of wound healing in diabetes. J Clin Invest.

[24] Giurato L, Meloni M, Izzo V, Uccioli L (2017). Osteomyelitis in diabetic foot: a comprehensive overview. World J Diabetes.

[25] Krauss JL, Roper PM, Ballard A (2019). *Staphylococcus aureus* infects osteoclasts and replicates intracellularly. MBio.

[26] Muthukrishnan G, Masters EA, Daiss JL (2019). Mechanisms of immune evasion and bone tissue colonization that make *Staphylococcus aureus* the primary pathogen in osteomyelitis. Curr Osteoporos Rep.

[27] Holtfreter S, Kolata J, Broker BM (2010). Towards the immune proteome of *Staphylococcus aureus*— the anti-*S. aureus* antibody response. Int J Med Microbiol.

[28] Pauli NT, Kim HK, Falugi F (2014). *Staphylococcus aureus* infection induces protein A mediated immune evasion in humans. J Exp Med.

[29] Suligoy CM, Lattar SM, Llana MN (2018). Mutation of Agr is associated with the adaptation of *Staphylococcus aureus* to the host during chronic osteomyelitis. Front Cell Infect Microbiol.

[30] Ramirez AM, Byrum SD, Beenken KE (2020). Exploiting correlations between protein abundance and the functional status of saeRS and sarA to identify virulence factors of potential importance in the pathogenesis of *Staphylococcus aureus* osteomyelitis. ACS Infect Dis.

[31] Víquez-Molina G, Aragón-Sánchez J, Pérez-Corrales C (2018). Virulence factor genes in *Staphylococcus aureus* isolated from diabetic foot soft tissue and bone infections. Int J Low Extrem Wounds.

[32] Lattar SM, Tuchscherr LPN, Centrón D (2012). Molecular fingerprinting of *Staphylococcus aureus* isolated from patients with osteomyelitis in Argentina and clonal distribution of the cap5(8) genes and of other selected virulence genes. Eur J Clin Microbiol Infect Dis.

[33] Senneville E, Briere M, Neut C (2014). First report of the predominance of clonal complex 398 *Staphylococcus aureus* strains in osteomyelitis complicating diabetic foot ulcers: a national French study. Clin Microbiol Infect.

[34] Otto M (2014). *Staphylococcus aureus* toxins. Curr Opin Microbiol.

[35] Xiong YQ, Willard J, Yeaman MR (2006). Regulation of *Staphylococcus aureus* α-toxin gene (hla) expression by agr, sarA and sae *in vitro* and in experimental infective endocarditis. J Infect Dis.

[36] Valeva A, Walev I, Pinkernell M (1997). Transmembrane β-barrel of staphylococcal α-toxin forms in sensitive but not in resistant cells. Proc Natl Acad Sci.

[37] Sotto A, Richard JL, Messad N (2012). Distinguishing colonization from infection with *Staphylococcus aureus* in diabetic foot ulcers with miniaturized oligonucleotide arrays: a French multicenter study. Diabetes Care.

[38] Djahmi N, Messad N, Nedjai S (2013). Molecular epidemiology of *Staphylococcus aureus* strains isolated from inpatients with infected diabetic foot ulcers in an Algerian University Hospital. Clin Microbiol Infect.

[39] Harch SAJ, MacMorran E, Tong SYC (2017). High burden of complicated skin and soft tissue infections in the indigenous population of Central Australia due to dominant Panton Valentine leucocidin clones ST93-MRSA and CC121-MSSA. BMC Infect Dis.

[40] Jauneikaite E, Ferguson T, Mosavie M (2020). *Staphylococcus aureus* colonization and acquisition of skin and soft tissue infection among royal marines recruits: a prospective cohort study. Clin Microbiol Infect.

[41] Balakirski G, Hischebeth G, Altengarten J (2020). Recurrent mucocutaneous infections caused by PVL-positive *Staphylococcus aureus* strains: a challenge in clinical practice. J Ger Soc Dermatol.

[42] Lina G, Piemont Y, Godail-Gamot F (1999). Involvement of Panton-Valentine leukocidin-producing *Staphylococcus aureus* in primary skin infections and pneumonia. Clin Infect Dis.

[43] Vazquez V, Liang X, Horndahl JK (2011). Fibrinogen is a ligand for the *Staphylococcus aureus* microbial surface components recognizing adhesive matrix molecules (MSCRAMM) bone sialoprotein-binding protein (Bbp). J Biol Chem.

[44] Persson L, Johansson C, Ryden C (2009). Antibodies to *Staphylococcus aureus* bone sialoprotein-binding protein indicate infectious osteomyelitis. Clin Vaccine Immunol.

[45] O’Riordan K, Lee JC (2004). *Staphylococcus aureus* capsular polysaccharides. Clin Microbiol Rev.

[46] Johansson A, Flock J-I, Svensson O (2001). Collagen and fibronectin binding in experimental staphylococcal osteomyelitis. Clin Orthop Relat Res.

[47] Patti JM, Bremell T, Krajewska-Pietrasik D (1994). The *Staphylococcus aureus* collagen adhesin is a virulence determinant in experimental septic arthritis. Infect Immun.

[48] Elasri MO, Thomas JR, Skinner RA (2002). *Staphylococcus aureus* collagen adhesin contributes to the pathogenesis of osteomyelitis. Bone.

[49] Rhem MN, Lech EM, Patti JM (2000). The collagen-binding adhesin is a virulence factor in *Staphylococcus aureus* keratitis. Infect Immun.

[50] Nethercott C, Mabbett AN, Totsika M (2013). Molecular characterization of endocarditis-associated *Staphylococcus aureus*. J Clin Microbiol.

[51] Liew YK, Hamat RA, van Belkum A, Chong PP, Neela V (2015). Comparative exoproteomics and host inflammatory response in *Staphylococcus aureus* skin and soft tissue infections, bacteremia, and subclinical colonization. Clin Vaccine Immunol.

[52] Corrigan RM, Miajlovic H, Foster TJ (2009). Surface proteins that promote adherence of *Staphylococcus aureus* to human desquamated nasal epithelial cells. BMC Microbiol.

[53] Sabat A, Melles DC, Martirosian G, Grundmann H, van Belkum A, Hryniewicz W (2006). Distribution of the serine-aspartate repeat protein-encoding sdr genes among nasal-carriage and invasive *Staphylococcus aureus* strains. J Clin Microbiol.

[54] Askarian F, Ajayi C, Hanssen A-M (2016). The interaction between *Staphylococcus aureus* SdrD and desmoglein 1 is important for adhesion to host cells. Sci Rep.

[55] Trad S, Allignet J, Frangeul L (2004). DNA macroarray for identification and typing of *Staphylococcus aureus* isolates. J Clin Microbiol.

[56] Sharp JA, Echague CG, Hair PS (2012). *Staphylococcus aureus* surface protein SdrE binds complement regulator factor H as an immune evasion tactic. PLoS One.

[57] Tung H, Guss B, Hellman U, Persson L, Rubin K, Ryden C (2000). A bone sialoprotein-binding protein from *Staphylococcus aureus*: a member of the staphylococcal Sdr family. Biochem J.

[58] Lemichez E, Lecuit M, Nassif X (2010). Breaking the wall: targeting of the endothelium by pathogenic bacteria. Nat Rev Microbiol.

[59] Aktories K (2011). Bacterial protein toxins that modify host regulatory GTPases. Nat Rev Microbiol.

[60] Boyer L, Doye A, Rolando M (2006). Induction of transient macroapertures in endothelial cells through RhoA inhibition by *Staphylococcus aureus* factors. J Cell Biol.

[61] Munro P, Benchetrit M, Nahori MA (2010). The *Staphylococcus aureus* epidermal cell differentiation inhibitor toxin promotes formation of infection foci in a mouse model of bacteremia. Infect Immun.

[62] Reyes-Robles T, Alonzo F, Kozhaya L, Lacy DB, Unutmaz D, Torres VJ (2013). *Staphylococcus aureus* leukotoxin ED targets the chemokine receptors CXCR1 and CXCR2 to kill leukocytes and promote infection. Cell Host Microbe.

[63] Alonzo F, Kozhaya L, Rawlings SA (2013). CCR5 is a receptor for *Staphylococcus aureus* leukotoxin ED. Nature.

[64] Labandeira-Rey M, Couzon F, Boisset S (2007). *Staphylococcus aureus* Panton-Valentine leukocidin causes necrotizing pneumonia. Science.

[65] Spaan AN, Henry T, Van Rooijen WJM (2013). The staphylococcal toxin Panton-Valentine leukocidin targets human C5a receptors. Cell Host Microbe.

[66] Diep BA, Sensabaugh GF, Somboona NS, Carleton HA, Perdreau-Remington F (2004). Widespread skin and soft-tissue infections due to two methicillin-resistant *Staphylococcus aureus* strains harboring the genes for Panton-Valentine leucocidin. J Clin Microbiol.

[67] Genestier AL, Michallet MC, Prevost G (2005). *Staphylococcus aureus* Panton-Valentine leukocidin directly targets mitochondria and induces Bax-independent apoptosis of human neutrophils. J Clin Invest.

[68] Vu BG, Stach CS, Salgado-Pabon W (2014). Superantigens of *Staphylococcus aureus* from patients with diabetic foot ulcers. J Infect Dis.

[69] Spaulding AR, Salgado-Pabón W, Kohler PL (2013). Staphylococcal and streptococcal superantigen exotoxins. Clin Microbiol Rev.

[70] Sotto A, Lina G, Richard J-L (2008). Virulence potential of *Staphylococcus aureus* strains isolated from diabetic foot ulcers: a new paradigm. Diabetes Care.

[71] Rolando M, Munro P, Stefani C (2009). Injection of *Staphylococcus aureus* EDIN by the *Bacillus anthracis* protective antigen machinery induces vascular permeability. Infect Immun.

[72] Messad N, Landraud L, Canivet B (2013). Distribution of edin in *Staphylococcus aureus* isolated from diabetic foot ulcers. Clin Microbiol Infect.

[73] Ji G, Beavis R, Novick RP (1997). Bacterial interference caused by autoinducing peptide variants. Science.

[74] Thompson TA, Brown PD (2017). Association between the agr locus and the presence of virulence genes and pathogenesis in *Staphylococcus aureus* using a *Caenorhabditis elegans* model. Int J Infect Dis.

[75] Diep BA, Gill SR, Chang RF (2008). Complete genome sequence of USA300, an epidemic clone of community-acquired meticillin-resistant *Staphylococcus aureus*. Lancet.

[76] Diep BA, Otto M (2008). The role of virulence determinants in community-associated MRSA pathogenesis. Trends Microbiol.

[77] Diep BA, Stone GG, Basuino L (2008). The arginine catabolic mobile element and staphylococcal chromosomal cassette mec linkage: convergence of virulence and resistance in the USA300 clone of methicillin-resistant *Staphylococcus aureus*. J Infect Dis.

[78] Montgomery CP, Boyle-Vavra S, Daum RS (2009). The arginine catabolic mobile element is not associated with enhanced virulence in experimental invasive disease caused by the community-associated methicillin-resistant *Staphylococcus aureus* USA300 genetic background. Infect Immun.

[79] Thurlow LR, Joshi GS, Clark JR (2013). Functional modularity of the arginine catabolic mobile element contributes to the success of USA300 methicillin-resistant *Staphylococcus aureus*. Cell Host Microbe.

[80] Planet PJ, LaRussa SJ, Dana A (2013). Emergence of the epidemic methicillin-resistant strain USA300 coincides with horizontal transfer of the arginine catabolic mobile element and speG-mediated *Staphylococcus aureus* adaptations for survival on skin. MBio.

[81] Cunningham R, Cockayne A, Humphreys H (1996). Clinical and molecular aspects of the pathogenesis of *Staphylococcus aureus* bone and joint infections. J Med Microbiol.

[82] Foster TJ (2019). The MSCRAMM family of cell-wall-anchored surface proteins of Gram-positive cocci. Trends Microbiol.

[83] Proctor RA, Von Eiff C, Kahl BC (2006). Small colony variants: a pathogenic form of bacteria that facilitates persistent and recurrent infections. Nat Rev Microbiol.

[84] Tuchscherr L, Heitmann V, Hussain M (2010). *Staphylococcus aureus* small-colony variants are adapted phenotypes for intracellular persistence. J Infect Dis.

[85] von Eiff C, Becker K, Metze D (2001). Intracellular persistence of *Staphylococcus aureus* small-colony variants within keratinocytes: a cause for antibiotic treatment failure in a patient with Darier’s disease. Clin Infect Dis.

[86] Garcia LG, Lemaire S, Kahl BC (2013). Antibiotic activity against small-colony variants of *Staphylococcus aureus*: review of *in vitro*, animal and clinical data. J Antimicrob Chemother.

[87] Dunyach-Remy C, Essebe CN, Sotto A, Lavigne JP (2016). *Staphylococcus aureus* toxins and diabetic foot ulcers: role in pathogenesis and interest in diagnosis. Toxins.

[88] Mongodin E, Bajolet O, Cutrona J (2002). Fibronectin-binding proteins of *Staphylococcus aureus* are involved in adherence to human airway epithelium. Infect Immun.

[89] Wang R, Braughton KR, Kretschmer D (2007). Identification of novel cytolytic peptides as key virulence determinants for community-associated MRSA. Nat Med.

[90] Zeng P, Xu C, Cheng Q (2019). Phenol-soluble-modulin-inspired amphipathic peptides have bactericidal activity against multidrug-resistant bacteria. ChemMedChem.

[91] Haggar A, Hussain M, Lönnies H (2003). Extracellular adherence protein from *Staphylococcus aureus* enhances internalization into eukaryotic cells. Infect Immun.

[92] Athanasopoulos AN, Economopoulou M, Orlova VV (2006). The extracellular adherence protein (Eap) of *Staphylococcus aureus* inhibits wound healing by interfering with host defense and repair mechanisms. Blood.

[93] Chavakis T, Hussain M, Kanse SM (2002). *Staphylococcus aureus* extracellular adherence protein serves as anti-inflammatory factor by inhibiting the recruitment of host leukocytes. Nat Med.

[94] Bur S, Preissner KT, Herrmann M, Bischoff M (2013). The *Staphylococcus aureus* extracellular adherence protein promotes bacterial internalization by keratinocytes independent of fibronectin-binding proteins. J Invest Dermatol.

[95] Vlassova N, Han A, Zenilman JM (2011). New horizons for cutaneous microbiology: the role of biofilms in dermatological disease. Br J Dermatol.

[96] Cooper RA, Bjarnsholt T, Alhede M (2014). Biofilms in wounds: a review of present knowledge. J Wound Care.

[97] Marano RJ, Wallace HJ, Wijeratne D (2015). Secreted biofilm factors adversely affect cellular wound healing responses *in vitro*. Sci Rep.

[98] Kirker KR, Secor PR, James GA, Fleckman P, Olerud JE, Stewart PS (2009). Loss of viability and induction of apoptosis in human keratinocytes exposed to *Staphylococcus aureus* biofilms *in vitro*. Wound Repair Regen.

[99] Tankersley A, Frank MB, Bebak M (2014). Early effects of *Staphylococcus aureus* biofilm secreted products on inflammatory responses of human epithelial keratinocytes. J Inflamm (Lond).

[100] Mottola C, Semedo-Lemsaddek T, Mendes JJ (2016). Molecular typing, virulence traits and antimicrobial resistance of diabetic foot staphylococci. J Biomed Sci.

[101] Feil EJ, Cooper JE, Grundmann H, Robinson DA, Enright MC, Berendt T (2003). How clonal is *Staphylococcus aureus*?. J Bacteriol.

[102] Kolawole DO, Adeyanju A, Schaumburg F (2013). Characterization of colonizing *Staphylococcus aureus* isolated from surgical wards’ patients in a Nigerian university hospital. PLoS One.

[103] Wehrhahn MC, Robinson JO, Pascoe EM (2012). Illness severity in community-onset invasive *Staphylococcus aureus* infection and the presence of virulence genes. J Infect Dis.

[104] Xiong YQ, Fowler VG, Yeaman MR, Perdreau-Remington F, Kreiswirth BN, Bayer AS (2009). Phenotypic and genotypic characteristics of persistent methicillin-resistant *Staphylococcus aureus* bacteremia *in vitro* and in an experimental endocarditis model. J Infect Dis.

[105] Egyir B, Guardabassi L, Sorum M (2014). Molecular epidemiology and antimicrobial susceptibility of clinical *Staphylococcus aureus* from healthcare institutions in Ghana. PLoS One.

[106] Menegotto F, Gonzalez-Cabrero S, Cubero A (2012). Clonal nature and diversity of resistance, toxins and adhesins genes of meticillin-resistant *Staphylococcus aureus* collected in a Spanish hospital. Infect Genet Evol.

[107] Xiao M, Zhao R, Zhang Q (2016). Genotypic diversity of *Staphylococcus aureus* α-hemolysin gene (*hla*) and its association with clonal background: implications for vaccine development. PLoS One.

[108] Ruzickova V, Pantucek R, Petras P, Machova I, Kostylkova K, Doskar J (2012). Major clonal lineages in impetigo *Staphylococcus aureus* strains isolated in Czech and Slovak maternity hospitals. Int J Med Microbiol.

[109] Shittu AO, Oyedara O, Okon K (2015). An assessment on DNA microarray and sequence-based methods for the characterization of methicillin-susceptible *Staphylococcus aureus* from Nigeria. Front Microbiol.

[110] Lozano C, Porres-Osante N, Crettaz J (2013). Changes in genetic lineages, resistance, and virulence in clinical methicillin-resistant *Staphylococcus aureus* in a Spanish hospital. J Infect Chemother.

[111] Tokajian ST, Khalil PA, Jabbour D (2010). Molecular characterization of *Staphylococcus aureus* in Lebanon. Epidemiol Infect.

[112] Luedicke C, Slickers P, Ehricht R, Monecke S (2010). Molecular fingerprinting of *Staphylococcus aureus* from bone and joint infections. Eur J Clin Microbiol Infect Dis.

[113] van Trijp MJCA, Melles DC, Snijders SV (2010). Genotypes, superantigen gene profiles, and presence of exfoliative toxin genes in clinical methicillin-susceptible *Staphylococcus aureus* isolates. Diagn Microbiol Infect Dis.

[114] Peacock SJ, Moore CE, Justice A (2002). Virulent combinations of adhesin and toxin genes in natural populations of *Staphylococcus aureus*. Infect Immun.

[115] Li S, Sun J, Zhang J (2014). Comparative analysis of the virulence characteristics of epidemic methicillin–resistant *Staphylococcus aureus* (MRSA) strains isolated from Chinese children: ST59 MRSA highly expresses core gene–encoded toxin. Apmis.

[116] Croes S, Deurenberg RH, Boumans MLL, Beisser PS, Neef C, Stobberingh EE (2009). *Staphylococcus aureus* biofilm formation at the physiologic glucose concentration depends on the *S. aureus* lineage. BMC Microbiol.

[117] Blomfeldt A, Eskesen AN, Aamot HV, Leegaard TM, Bjornholt JV (2016). Population-based epidemiology of *Staphylococcus aureus* bloodstream infection: clonal complex 30 genotype is associated with mortality. Eur J Clin Microbiol Infect Dis.

[118] Cheung GYC, Kretschmer D, Duong AC (2014). Production of an attenuated phenol-soluble modulin variant unique to the MRSA clonal complex 30 increases severity of bloodstream infection. PLoS Pathog.

[119] Rieg S, Jonas D, Kaasch AJ (2013). Microarray-based genotyping and clinical outcomes of *Staphylococcus aureus* bloodstream infection: an exploratory study. PLoS One.

[120] Wu D, Li X, Yang Y (2011). Superantigen gene profiles and presence of exfoliative toxin genes in community-acquired meticillin-resistant *Staphylococcus aureus* isolated from Chinese children. J Med Microbiol.

[121] Ghasemzadeh-Moghaddam H, Ghaznavi-Rad E, Sekawi Z (2011). Methicillin-susceptible *Staphylococcus aureus* from clinical and community sources are genetically diverse. Int J Med Microbiol.

[122] Yamaguchi T, Okamura S, Miura Y, Koyama S, Yanagisawa H, Matsumoto T (2015). Molecular characterization of community-associated methicillin-resistant *Staphylococcus aureus* isolated from skin and pus samples of outpatients in Japan. Microb Drug Resist.

[123] Stieber B, Monecke S, Muller E, Baier V, Coombs GW, Ehricht R (2014). Development and usage of protein microarrays for the quantitative measurement of Panton-Valentine leukocidin. Mol Cell Probes.

[124] Bennett CM, Coombs GW, Wood GM (2014). Community-onset *Staphylococcus aureus* infections presenting to general practices in South-eastern Australia. Epidemiol Infect.

[125] Shore AC, Tecklenborg SC, Brennan GI, Ehricht R, Monecke S, Coleman DC (2014). Panton-Valentine leukocidin-positive *Staphylococcus aureus* in Ireland from 2002 to 2011: 21 clones, frequent importation of clones, temporal shifts of predominant methicillin-resistant *S. aureus* clones, and increasing multiresistance. J Clin Microbiol.

[126] Chen L, Mediavilla JR, Smyth DS (2010). Identification of a novel transposon (Tn6072) and a truncated staphylococcal cassette chromosome mec element in methicillin-resistant *Staphylococcus aureus* ST239. Antimicrob Agents Chemother.

[127] Deurenberg RH, Rijnders MIA, Sebastian S, Welling MA, Beisser PS, Stobberingh EE (2009). The *Staphylococcus aureus* lineage-specific markers collagen adhesin and toxic shock syndrome toxin 1 distinguish multilocus sequence typing clonal complexes within spa clonal complexes. Diagn Microbiol Infect Dis.

[128] Monecke S, Müller E, Büchler J, Stieber B, Ehricht R (2014). Staphylococcus aureus in vitro secretion of alpha toxin (hla) correlates with the affiliation to clonal complexes. PLoS One.

[129] Schmidt T, Zündorf J, Grüger T (2013). Phenotyping of *Staphylococcus aureus* reveals a new virulent ST398 lineage. Clin Microbiol Infect.

[130] Fitzgerald JR (2012). Livestock-associated *Staphylococcus aureus*: origin, evolution and public health threat. Trends Microbiol.

[131] McCarthy AJ, Van Wamel W, Vandendriessche S (2012). *Staphylococcus aureus* CC398 clade associated with human-to-human transmission. Appl Environ Microbiol.

[132] Witte W, Strommenger B, Stanek C, Cuny C (2007). Methicillin-resistant *Staphylococcus aureus* ST398 in humans and animals, central Europe. Emerg Infect Dis.

[133] Kock R, Schaumburg F, Mellmann A (2013). Livestock-associated methicillin-resistant *Staphylococcus aureus* (MRSA) as causes of human infection and colonization in Germany. PLoS One.

[134] Zhao C, Liu Y, Zhao M (2012). Characterization of community acquired *Staphylococcus aureus* associated with skin and soft tissue infection in Beijing: high prevalence of PVL+ ST398. PLoS One.

[135] Price LB, Stegger M, Hasman H (2012). *Staphylococcus aureus* CC398: host adaptation and emergence of methicillin resistance in livestock. MBio.

[136] Li M, Cheung GYC, Hu J (2010). Comparative analysis of virulence and toxin expression of global community-associated methicillin-resistant *Staphylococcus aureus* strains. J Infect Dis.

[137] Lipsky BA, Aragon-Sanchez J, Embil J (2016). International Working Group on the Diabetic Foot (IWGDF). IWGDF guidance on the diagnosis and management of foot infections in persons with diabetes. Diabetes Metab Res Rev.

[138] Lipsky BA, Senneville É, Abbas ZG (2020). Guidelines on the diagnosis and treatment of foot infection in persons with diabetes (IWGDF 2019 update). Diabetes Metab Res Rev.

[139] Post V, Wahl P, Uckay I (2014). Phenotypic and genotypic characterisation of *Staphylococcus aureus* causing musculoskeletal infections. Int J Med Microbiol.

[140] Messad N, Prasjnar TK, Lina G (2015). Existence of a colonizing *Staphylococcus aureus* strain isolated in diabetic foot ulcers. Diabetes.

